# From Brudzinski to Jamil: Unveiling Classical and Emerging Clinical Signs of Meningitis

**DOI:** 10.7759/cureus.93196

**Published:** 2025-09-25

**Authors:** Mamoon Khan, Muhammad Shakeel, Muhammad Salman, Zarshal Zakir, Ahmad Hashmat, Saira K Awan, Giustino Varrassi

**Affiliations:** 1 Diabetes and Endocrinology, University Hospitals of Derby and Burton NHS Foundation Trust, Derby, GBR; 2 Medicine and Surgery, Khyber Medical College, Peshawar, PAK; 3 Acute Medicine, Blackpool Teaching Hospitals NHS Foundation Trust, Blackpool, GBR; 4 Acute Medicine, Queen Elizabeth Hospital Birmingham, Birmingham, GBR; 5 Family Medicine, CHI St. Alexius Health, Bismarck, USA; 6 Nephrology, Richmond University Medical Center, Staten Island, USA; 7 Neurology, Advanced Neurology P.C., Brooklyn, USA; 8 Medicine, Kabir Medical College, Peshawar, PAK; 9 Pain Medicine, Fondazione Paolo Procacci, Rome, ITA

**Keywords:** fever, meningeal irritation, meningitis, neck stiffness, photophobia

## Abstract

Meningitis can result from bacterial, viral, fungal, or noninfectious causes and requires early recognition to reduce both death and long-term complications. While laboratory testing and imaging confirm the diagnosis, bedside examination remains pivotal in guiding initial clinical decisions. This narrative review summarizes the main clinical signs of meningitis, both classical and more recently described, and discusses their diagnostic value. A structured literature search was conducted across major medical databases and sources, focusing on physical signs rather than laboratory or imaging approaches. Classic signs such as Kernig’s and Brudzinski’s are well recognized but limited by variable sensitivity. Other bedside maneuvers, including jolt accentuation and less commonly used signs, provide additional diagnostic context. Emerging signs, such as Jamil’s sign, highlight the potential for improved detection of subtle meningeal irritation. Although no single sign can confirm or exclude meningitis with certainty, combining several findings with careful clinical judgment enhances diagnostic accuracy. In resource limited settings, bedside signs remain especially critical where access to imaging and laboratory confirmation may be delayed. Training clinicians in the correct interpretation and performance of these signs, and incorporating these into practice, can improve patient outcomes while strengthening early diagnosis. Future work should continue to validate emerging signs across diverse populations and encourage uniform examination protocols.

## Introduction and background

Meningitis occurs when the protective layers around the brain and spinal cord, known as the meninges, become inflamed. This inflammation can be triggered by a range of causes, including bacterial, viral, or fungal infections, as well as noninfectious factors such as certain medicines, autoimmune conditions, or cancers. The condition often develops rapidly and may present with fever, headache, stiff neck, and altered mental status. Because the disease can progress quickly, fast diagnosis and treatment are critical. Even short delays in care can result in severe complications such as seizures, hearing loss, neurological damage, or death [1-4].

Laboratory tests, such as cerebrospinal fluid (CSF) analysis, blood cultures, and imaging studies, are essential for confirming meningitis and identifying its cause. However, because the disease can progress quickly, clinicians often begin treatment before definitive results are available. In these cases, a careful physical examination becomes critical, as certain bedside signs can raise suspicion and prompt urgent action. This is especially important in resource-limited settings where advanced diagnostic tools may be limited or unavailable, making clinical assessment the primary method for early detection and management [5].

Doctors have long relied on Kernig's and Brudzinski's signs to detect meningeal irritation during physical examination. These maneuvers can be helpful when positive, as they indicate stretching of the inflamed meninges. However, studies show that most patients with meningitis never demonstrate these signs, limiting their diagnostic value in practice. This gap highlights the need for better bedside tests that are more sensitive and reliable, especially in situations where immediate decisions are critical and advanced diagnostics are not yet available [6].

Over the years, new signs have emerged. Jolt accentuation of headache can flag meningitis when classic signs are absent [7,8]. Tripod and Amoss's signs hint at severe spinal irritation in children. Opisthotonos signals advanced disease but appears late. Each adds value, yet none alone suffice [9].

In 2022, Jamil's sign entered the scene. It tests neck rigidity with the patient lying on their side. Early studies report very high sensitivity and specificity. This sign may help catch cases that other tests miss [10].

This review brings together all major physical signs of meningitis. We explain how each test is performed and discuss its strengths and limitations. We highlight practical tips for varied patient groups, from infants to the elderly. Our goal is to help spot meningitis quickly and act before confirmatory tests return.

## Review

Methodology

We conducted a narrative review to gather and summarize key clinical signs of meningitis. We structured our work around the Scale for the Assessment of Narrative Review Articles criteria to keep the process clear, rigorous, and relevant [11].

Search Strategy

We searched PubMed, MEDLINE, EMBASE, Google Scholar, Scopus, and the Cochrane Library for studies published from 1990 to 2025. We combined terms such as “meningitis”, “clinical signs”, “Brudzinski”, “Kernig”, “Jamil sign”, “neck stiffness”, and “neurological exam”. We also reviewed the WHO and CDC guidelines by hand to capture any additional recommendations.

Inclusion and Exclusion

We included English-language original research, reviews, case series, expert guidelines, and consensus documents that described bedside physical signs of meningitis across age groups. We excluded papers focused only on lab or imaging diagnostics, non-English publications, non-peer-reviewed articles, and studies limited to neonates unless their signs applied broadly.

Data Extraction and Synthesis

For each eligible study, we pulled details on how to perform the sign, its diagnostic accuracy, its practical use, and its limitations. We then grouped signs into classic (Kernig's and Brudzinski's) versus emerging or lesser known (jolt accentuation, tripod/Amoss's, and Jamil's signs). Our narrative highlights which signs work best for early detection, especially in settings without prompt access to lab tests or imaging.

Classical clinical features

Fever

Fever is a near-universal symptom of meningitis across all age groups. High-grade fever (102.4°F/39.1°C or higher) is more typical in acute bacterial meningitis, whereas viral or subacute meningitis may cause lower grade fever (between 99.1°F/37.3°C and 100.4°F/38°C) [1-4]. However, the absence of fever does not rule out meningitis, especially in vulnerable populations. Elderly or immunocompromised patients may present with no fever or only mild fever due to blunted immune responses. Fever should always be interpreted with caution in a high-risk patient [12].

Headache

An intense, diffuse, and persistent headache is a hallmark of meningitis. Patients often describe it as one of the worst headaches. The headache is typically aggravated by movements that increase intracranial pressure (ICP) [13]. Bending over, coughing, or sneezing can worsen the pain. Photophobia (light sensitivity) commonly accompanies the headache due to meningeal irritation of the cranial nerves [14]. The presence of severe headache, especially with fever and neck stiffness, should raise a strong suspicion for meningitis [1].

Neck Stiffness (Nuchal Rigidity)

Resistance to passive neck flexion, or nuchal rigidity, is a classic sign of meningeal irritation [13]. On examination, the patient cannot comfortably flex the neck forward; attempting to do so causes pain and muscular spasm. Neck stiffness is highly specific for meningitis; if present, it strongly suggests meningeal inflammation, but it is unfortunately not very sensitive. Studies have shown that only about 15%-30% of meningitis patients (especially adults) exhibit nuchal rigidity on initial exam [15]. The yield is even lower in very young infants and in the elderly, where baseline neck mobility or neurologic status may limit the exam. Importantly, the absence of neck stiffness does not exclude meningitis, and clinical judgment must integrate this finding with the overall picture [16].

Photophobia and Phonophobia

Many patients with meningitis develop photophobia, an extreme sensitivity to light, due to irritation of the meninges and cranial nerves [13]. They may prefer dark environments and experience worsening headache or nausea in bright light. Phonophobia (sensitivity to loud sounds) can similarly occur. These symptoms are often reported subjectively and are more common in viral or “aseptic” meningitis, but they can appear with any meningitis. While not specific to meningitis, the combination of photophobia with fever and headache is concerning for meningeal inflammation [17,18].

Nausea and Vomiting

Nausea and vomiting frequently accompany meningitis, largely due to increased ICP and meningeal irritation triggering the vomiting center [13]. Vomiting in meningitis is often projectile and not preceded by significant nausea. These gastrointestinal symptoms may precede overt neurological signs and can sometimes lead clinicians astray. In the context of fever and headache, unexplained vomiting should prompt consideration of a central nervous system cause like meningitis [17,18].

Altered Mental Status

Changes in mental status range from mild lethargy or confusion to deep coma, depending on the severity and progression of meningitis. Patients may be difficult to arouse, disoriented, or irritable. This is more pronounced in bacterial meningitis, which can progress rapidly to obtundation. Viral meningitis patients are often alert and oriented (or only mildly lethargic), whereas tuberculous meningitis can cause subtle personality changes early on. Any acute confusion or decreased level of consciousness in a febrile patient should prompt evaluation for meningitis among other diagnoses. In fact, altered mental status is part of the classic triad in adults [1,13,18].

Special physical examination signs of meningeal irritation

Beyond the general symptoms above, clinicians have long relied on specific physical exam maneuvers to detect meningeal irritation. The most well-known are Kernig's sign and Brudzinski's sign, described over a century ago [6,19,20]. While these eponymous signs are highly specific for meningitis, they unfortunately have low sensitivity, meaning many confirmed meningitis cases will not exhibit them. Nonetheless, when present, they provide strong supportive evidence of the diagnosis. Several other signs (some historical, some new) can also point toward meningitis. Below is a review of these exam signs.

Kernig's sign

Kernig's sign is elicited with the patient lying supine. The examiner flexes the patient's hip to a 90° angle (hip and knee both flexed). Then, the examiner attempts to extend the leg at the knee. A positive Kernig's sign is when this maneuver causes pain in the lower back or resistance to straightening the leg. In practice, it is often the hamstring tightness and pain behind the thigh that limit the leg extension [21]. Passive knee extension in this position stretches the irritated meninges and spinal nerve roots. In meningitis, the inflamed meninges or nerve roots are painful when stretched. The resistance and pain are due to a reflex contraction of the hamstring muscles in response to this stretch. Essentially, a patient with meningeal inflammation will involuntarily guard against the maneuver to avoid traction on the sensitive meninges and roots [21].

Kernig's sign is highly specific for meningitis but has very low sensitivity (5%-27%). A positive Kernig's sign strongly suggests meningeal irritation, since few other conditions cause this exact finding. However, most patients with meningitis will not have a positive Kernig's sign. It tends to be more frequently positive in adolescents and young adults and in cases of severe bacterial meningitis. Specificity, on the other hand, is about 90%-95%; a positive Kernig's sign is rarely seen in illnesses other than meningitis. Because of its low sensitivity, a negative Kernig's sign never rules out meningitis. Kernig's sign is best used in combination with other signs and overall clinical judgment. When positive, it adds weight to the decision to perform a lumbar puncture (LP). When negative, one must rely on other findings [21].

Brudzinski's sign

Brudzinski's neck sign is tested with the patient supine. The examiner gently but firmly flexes the patient's neck by lifting the head toward the chest. A positive Brudzinski's sign is the involuntary flexion of the hips and knees in response to neck flexion. In other words, when the patient's neck is passively flexed, they reflexively draw their legs up (bend at the knees and hips). This was described by Josef Brudzinski in 1909 as a sign of meningitis [5].

Neck flexion stretches the spinal meninges, especially in the vertical axis. In a patient with meningeal inflammation, this stretch is painful and triggers a protective reflex. By flexing the hips and knees, the patient attempts to reduce tension on the meninges. Bending the legs relaxes some of the stretch on the lumbosacral nerve roots and meninges. It is an involuntary defensive reaction. The Brudzinski response can be quite subtle; sometimes even a slight hip flexion or a wince may be the only sign. In a fully positive Brudzinski, both legs will draw up toward the abdomen when the neck is flexed [5].

Like Kernig's, Brudzinski's sign is highly specific but not very sensitive for meningitis. A positive Brudzinski's sign strongly suggests meningeal irritation. The reported sensitivity ranges from about 5% in some adult series up to 50% in others. Specificity is quite high, around 90% or more. Thus, as with Kernig's, a positive Brudzinski's sign is very helpful to “rule in” meningitis, but its absence does not “rule out” much. Combining Brudzinski's and Kernig's signs slightly improves sensitivity (one or the other might be present). Overall, Brudzinski's sign is a useful bedside tool when positive, and it often accompanies a positive Kernig's sign in classic teaching [19-22].

Jolt accentuation of headache

Jolt accentuation is a bedside test first described in 1991 as a way to detect subtle meningitis, particularly in patients with headache. To perform the test, an alert patient with a baseline headache is asked to rapidly rotate their head horizontally (side to side) at a frequency of about two to three turns per second [7]. A positive jolt accentuation occurs if this rapid head turning markedly worsens the patient's headache. Essentially, the maneuver “jolts” the inflamed meninges and, if meningitis is present, will exacerbate the headache beyond its baseline level. If the headache does not worsen with the maneuver, the test is negative. This test requires that the patient is cooperative and able to report changes in headache severity, so it cannot be done in those with reduced consciousness [7].

The rapid head turning causes a relative displacement of CSF and stretches the meninges. In an inflamed state (meningitis), this jostling of the meninges triggers increased pain. It transiently raises ICP and causes friction along the meninges. In a normal state (no meningitis), moving the head side to side at that pace might be uncomfortable, but typically does not significantly worsen a headache [7,8].

Jolt accentuation of headache has been found to be a moderately sensitive and highly specific sign for detecting meningitis, especially aseptic (viral) meningitis. The original Japanese study by Uchihara and Tsukagoshi in 1991 reported a very high sensitivity of about 97% for CSF pleocytosis (in other words, almost all patients with meningitis had a positive jolt test) [7]. Specificity in that study was around 60%. Subsequent studies, however, have shown more variable performance. A meta-analysis (Iguchi et al) of jolt accentuation reported a pooled sensitivity of about 65% and a specificity of nearly 70% for acute meningitis. If a patient with fever and headache has a clearly positive jolt accentuation, it should prompt immediate LP evaluation. One should note that jolt accentuation is not applicable in children too young to cooperate, nor in patients with severely altered mental status. It is most valid in conscious, oriented patients with headache [8].

Tripod sign and Amoss's sign

The Tripod sign is an old pediatric exam observation for meningeal irritation. It refers to a child's characteristic posture when sitting; the child supports themselves with their arms extended behind them, leaning backward in a tripod-like fashion [6]. Amoss's sign is closely related, described as the patient needing to use their hands for support in transitioning from lying to sitting due to pain in the back. Essentially, both signs reflect the patient’s attempt to stabilize the trunk and minimize flexion of the spine. These signs are typically observed in children. For instance, a toddler with meningitis may refuse to sit up straight and instead prop themselves up with their arms extended backward, often with the spine slightly arched and neck extended, to alleviate discomfort [6,22].

Tripod/Amoss's sign arises from spinal and meningeal irritation, causing pain on trunk flexion. By leaning back on the arms, the patient reduces the need to flex the spine or neck. This posture indicates that sitting up straight is painful, likely due to the stretching of inflamed meninges around the spinal cord. In children with meningitis, any movement that jars the spine (such as sitting up) can cause pain, so they instinctively use their arms to brace and limit movement [6,22].

This sign is a historical pediatric sign that is not commonly documented in modern studies, but it can still be observed. Its main utility is in young children or infants who cannot verbalize their symptoms [6]. If a febrile child assumes a tripod posture, one should be concerned about meningeal irritation. A child with hands braced behind and a refusal to bend forward should raise suspicion of meningitis or spinal meningismus [22]. Tripod/Amoss's signs do not have quantified sensitivity/specificity data, but they are considered nonspecific (other conditions can cause a child to refuse sitting forward) and not very sensitive. These signs have largely fallen out of favor in adult medicine (adults will usually voice their neck/back pain rather than assume the posture). In pediatric populations, if these signs are noted, they are certainly meaningful [6,22].

Jamil's sign

Jamil's sign is a recently described physical exam sign (first reported in 2022) for meningitis. It involves assessing neck stiffness with the patient in the lateral decubitus position (lying on the side) rather than supine. To elicit Jamil's sign, place the patient in the lateral decubitus position. The examiner supports the patient's head, one hand under the occiput (back of the head) and the other hand under the chin. Then, the examiner fully extends the neck and then flexes the neck (essentially rocking the head backward and forward through a full range of motion). During this maneuver, the examiner gauges the neck muscle tone and resistance. If there is notable resistance, rigidity, or abrupt stiffness felt on either extension or flexion, then Jamil's sign is positive. If the neck moves freely (supple) with no increased tone, Jamil's sign is negative. The key is the lateral positioning and the examiner's hands providing good support to feel subtle changes in tone [10].

Jamil's sign is essentially another way to detect nuchal rigidity, but the lateral positioning offers some mechanical advantages. By placing the patient on their side, the examiner can use gravity and full support of the head to achieve a greater range of motion in the neck safely. The maneuver stretches the meninges similar to how classic neck flexion does, but the examiner may have finer control and can better appreciate resistance with their hands under the head and chin. The theory is that this method allows detection of even mild neck stiffness that might be missed with the crude supine flexion test [10].

The study found that Jamil's sign could be a highly sensitive and specific indicator of meningitis. In the initial prospective study (419 patients), Jamil's sign was present in 357 of 361 confirmed meningitis cases, and absent in 53 of 58 nonmeningitis cases. This corresponds to a sensitivity of 98.9% and specificity of 91.4%, which are remarkably high [10]. These numbers, if validated, would make Jamil's sign one of the best bedside tests for meningitis diagnosis. Such accuracy could help avoid unnecessary LPs in cases that turn out not to be meningitis. Importantly, the test was studied in a setting with a high pre-test probability (patients suspected of meningitis), which is similar to how it would be used clinically. A negative Jamil's sign in a patient with equivocal neck stiffness supine might reassure a clinician to look for other causes, whereas a positive Jamil’s sign would strongly push toward performing an LP [10]. Table 1 summarizes these physical signs of meningitis for easy comparison.

**Table 1:** Clinical signs of meningeal irritation CSF: cerebrospinal fluid; LP: lumbar puncture Source: [5-10,16-22]

Sign	How to elicit	Mechanism	Clinical utility	Sensitivity/specificity	Key limitations
Kernig’s sign	Patient supine; flex hip and knee to 90°, then extend knee. Positive: pain or resistance on knee extension	Stretching of the inflamed meninges and nerve roots causes hamstring spasm	Classic bedside sign. When positive, strongly suggests meningitis (high specificity)	Sensitivity: 5%-27%; specificity: 90%-95%	Often absent in infants, the elderly, or altered patients. Low sensitivity means a negative sign is common even in meningitis
Brudzinski’s sign	Patient supine; passively flex the neck. Positive: involuntary hip/knee flexion (legs draw up)	Neck flexion stretches the meninges, causing pain; the patient reflexively flexes their legs to reduce meningeal tension	Useful when clearly positive; indicates meningeal irritation. Often performed together with Kernig’s	Sensitivity: 5%-60%; specificity: 80%-90%	Unreliable in extremes of age (infants <6 months may not show it; elderly may have limited neck motion). Not present in many confirmed cases
Jolt accentuation	Patient (alert) turns head rapidly side to side at 2-3 turns/second. Positive: headache worsens during the maneuver	Rapid head rotation shifts CSF and stretches meninges; inflamed meninges respond with increased pain	Good screening test in awake patients with fever and headache, especially for detecting viral/aseptic meningitis early	Sensitivity: 60%-70%; specificity: 80%-90%	Requires patient cooperation (cannot use if lethargic or very young). A negative result does not fully exclude meningitis
Tripod/Amoss’s sign	Seen mainly in children: when sitting, the patient leans back on hands for support (tripod posture) or needs arms to push up from lying to sitting (Amoss’s)	Indicates spinal rigidity, sitting up stretches spinal meninges, so child avoids it by bracing with arms. Reduces flexion of the spine to alleviate pain	Mostly historical. If observed in a febrile child, strongly suggests meningeal irritation. Useful in nonverbal children as a clue	No modern data. Considered low sensitivity. Specificity is not well defined	Nonspecific (other illnesses or weakness can cause a child to use arms to sit up). Rarely used in adults. Often absent even in pediatric meningitis today
Jamil’s sign	Patient in lateral position; examiner supports head (one hand under occiput, one under chin) and flexes/extends neck fully. Positive: felt resistance or stiffness on either motion	Improved leverage in lateral decubitus allows detection of any neck tone increase. Irritated meninges cause palpable neck rigidity during flexion/extension	High diagnostic value. It can potentially identify meningitis in patients with otherwise equivocal neck exams. May reduce unnecessary LPs when negative	Sensitivity 98.9% and specificity 91.4% in the initial study	New sign, not yet widely verified beyond initial study. Clinicians need training to perform correctly

Limitations

It must be emphasized that no single physical sign is 100% reliable for diagnosing or excluding meningitis [16-22]. While newer signs like Jolt accentuation and Jamil's sign offer improved sensitivity, they are not infallible in isolation either, and Jamil’s sign, though extremely promising, awaits further validation [7-9].

Clinical judgment remains paramount. A constellation of findings, including fever, headache, any neck stiffness, and abnormal mentation, should prompt investigation for meningitis even if classic signs are negative. When suspicion is high, an LP should be performed regardless of physical exam findings, after appropriate precautions (like neuroimaging if indicated). Time is critical in meningitis, particularly bacterial: delays in treatment markedly worsen outcomes. Indeed, studies suggest that, in adults, the presence of any two of the four symptoms (headache, fever, neck stiffness, and altered sensorium) is enough to warrant an LP in suspected meningitis.

Another limitation is patient cooperation. Agitated or obtunded patients cannot adequately participate in some maneuvers (like jolt accentuation). Infants cannot report headache or photophobia. In such cases, one must go by observation (Does the infant cry when moved? Is there spontaneous neck retraction or arching?) and have a low threshold for further investigation.

It is also important to note that a physical exam cannot differentiate etiologies definitively; for instance, Kernig's sign does not identify whether it is bacterial or viral meningitis. That requires laboratory testing.

## Conclusions

Meningitis presents a diagnostic challenge due to its broad clinical spectrum and variable presentation across different age groups and immune statuses. While laboratory tests and imaging are critical for definitive diagnosis, clinical signs remain indispensable, particularly in acute and resource-limited settings. Features such as fever, headache, and neck stiffness remain foundational, but physical examination signs like Kernig's and Brudzinski's continue to offer valuable bedside clues despite their limited sensitivity. While each sign has its limitations, when taken together and interpreted in context, they often ring the alarm for meningitis even before confirmatory tests. These clinical signs can strengthen the clinical diagnosis of meningitis, guiding timely LP and treatment, which are vital for patient outcomes.
